# Characteristics of Henle’s fiber layer in healthy and glaucoma eyes assessed by polarization-sensitive optical coherence tomography

**DOI:** 10.1364/BOE.485327

**Published:** 2023-05-16

**Authors:** Alice R. Motschi, Florian Schwarzhans, Sylvia Desissaire, Stefan Steiner, Hrvoje Bogunović, Philipp K. Roberts, Clemens Vass, Christoph K. Hitzenberger, Michael Pircher

**Affiliations:** 1 Medical University of Vienna, Center for Medical Physics and Biomedical Engineering, Vienna, Austria; 2 Medical University of Vienna, Center for Medical Statistics, Informatics and Intelligent Systems, Vienna, Austria; 3 Medical University of Vienna, Department of Clinical Pharmacology, Vienna, Austria; 4 Medical University of Vienna, Department of Ophthalmology and Optometry, Vienna, Austria; 5 Medical University of Vienna, Christian Doppler Laboratory for Artificial Intelligence in Retina, Vienna, Austria

## Abstract

Using conventional optical coherence tomography (OCT), it is difficult to image Henle fibers (HF) due to their low backscattering potential. However, fibrous structures exhibit form birefringence, which can be exploited to visualize the presence of HF by polarization-sensitive (PS) OCT. We found a slight asymmetry in the retardation pattern of HF in the fovea region that can be associated with the asymmetric decrease of cone density with eccentricity from the fovea. We introduce a new measure based on a PS-OCT assessment of optic axis orientation to estimate the presence of HF at various eccentricities from the fovea in a large cohort of 150 healthy subjects. By comparing a healthy age-matched sub-group (N = 87) to a cohort of 64 early-stage glaucoma patients, we found no significant difference in HF extension but a slightly decreased retardation at about 2° to 7.5° eccentricity from the fovea in the glaucoma patients. This potentially indicates that glaucoma affects this neuronal tissue at an early state.

## 1. Introduction

The macula, including its central part, the fovea, is the location of best visual acuity in humans and plays a key role in our daily life that is to a large extent based on vision. The fovea contains the highest density of cone photoreceptors and anterior neuronal tissue is pushed aside during the developmental phase of this retinal region to provide optimal light conditions for the underlying photoreceptors [1]. As a result of this lateral displacement, photoreceptor axons that connect with the neuronal retinal tissue are elongated and referred to as Henle fibers [2]. These long cylindrical structures extend radially from the fovea and connect with bipolar and horizontal cells at a location that is several hundreds of micrometers displaced from the fovea with an average length of Henle fibers of about 560 µm [3]. In this histological study, the extension of Henle’s fiber layer (HFL) was found to be up to 3.4 mm (nasally) and 4.5 mm (temporally) from the foveal center [3]. A recent in-vivo study that was based on averaged directional OCT scans used thickness measurements of HFL to estimate the extension of HFL [4]. Thereby, the authors reported an area of HFL that is much smaller as compared to the data of the histological study.

**Fig. 1.**
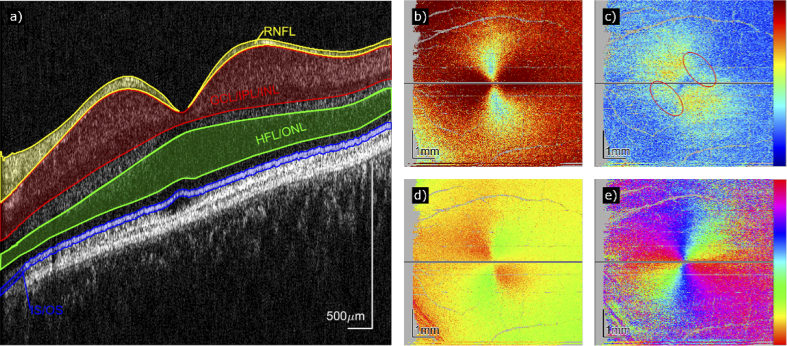
Intensity B-scan (a) and en-face maps of PS data (b–e) of a 30-year old male healthy volunteer. (a) The location of the intensity B-scan is indicated by a horizontal black line in (b)–(e). Yellow: RNFL (retinal nerve fiber layer), a birefringent structure. Red: GCL (ganglion cell layer), IPL (inner plexiform layer), and INL (inner nuclear layer): area where the brightest pixel per A-scan was used for the compensation of anterior birefringent structures. Green: HFL (Henle’s fiber layer) and ONL (outer nuclear layer). Blue: IS/OS (boundary between inner and outer photoreceptor segments): location where the PS data was retrieved. (b) Uncompensated retardation measured at IS/OS. (c) Retardation compensated for anterior birefringent structures but before final correction. The area marked by red ellipses indicates an incomplete compensation. (d) Uncompensated axis orientation measured at IS/OS. (e) Axis orientation compensated for anterior birefringent structures before final correction. *Colorbars: Retardation: 0° to 25°. Axis orientation: −90° to 90°*.

Due to the specific structure of HF, backscattering of light from these fibers strongly depends on the incidence angle of the light beam in respect to the fiber orientation. Strong backscattering can only be observed when the incidence angle of the imaging beam is close to 90°. Thus, in cross-sectional images that have been recorded with optical coherence tomography (OCT), typically a low signal can be observed from HFL (contained within the green area in Fig. 1(a)) [5]. Only a change in the incidence angle results in an increase of backscattered light from Henle’s fiber layer and thus leads to an increased signal strength in OCT images [4,5]. This condition can either be introduced intentionally by using an imaging beam that enters the eye off the central pupil location or is introduced by a deterioration of the normal retinal structure caused by pathology.

An additional aspect of fibrous structures such as HFL or retinal nerve fiber layer (RNFL) is the associated optical property of form birefringence [6]. In combination with polarized light the presence of birefringence within HFL results in optical effects that can be observed in the macula [7–9]. Using polarization-sensitive imaging methods such as scanning laser polarimetry (SLP) [10] or polarization-sensitive optical coherence tomography (PS-OCT) [11], this tissue-specific property can be exploited to gain additional information on HFL and RNFL. This is specifically relevant for observations of HFL as this layer shows only low signal strength in intensity based OCT. As the anterior eye segment (mainly the cornea) is birefringent as well [12], a combined effect on the backscattered polarized light is observed in the macula giving rise to an hour glass pattern in en-face retardation images (cf. Figure 1(b)) that originate from the posterior retinal layers (mainly the photoreceptor layers) [8,13]. This pattern is used in SLP to determine and to compensate for the influence of the anterior segment on polarization-sensitive measurements of the retinal nerve fiber layer [8] and to determine the quality of corneal compensation in PS-OCT [14].

After compensating for anterior segment birefringence, a “donut-shaped” phase retardation pattern in the macula of the healthy eye can be observed (cf. Fig. 1(c)) [8,14–16]. Alterations of this pattern may be associated to photoreceptor loss and it could be shown that the phase retardation of Henle fibers is reduced in patients with age-related macular degeneration (AMD) as compared to healthy eyes [17]. Similarly, poor visual acuity in neovascular AMD was linked to a disrupted Henle pattern [18] and it could be shown that foveal retardation was altered in elderly healthy volunteers, indicating structural changes of HFL due to normal aging [19].

A first quantitative assessment of the birefringent properties of HFL using PS-OCT was presented by Cense et al. [16]. Elevated retardation was found in an annulus around the fovea with a maximum (double pass) phase retardation of 20° to 25° and located on average at 1.8° eccentricity from the fovea [16]. The measured fast axis orientation which is related to the fiber orientation of HFL showed a radial pattern close to the fovea (as expected from histology), but was not visible in the periphery of the field of view that covered in this work the central 15° [16]. One additional aspect is that their pioneering study was limited to three healthy volunteers.

In this paper, we investigate HFL using PS-OCT in a large cohort of 150 healthy volunteers. We introduce a new measure to determine the presence of these fibers at various eccentricities from the fovea and provide quantitative data on the polarization properties of HFL. Finally, we compare for our Henle fiber analysis the PS-OCT retrieved data of 87 age-matched healthy subjects with data from 64 glaucoma patients.

## 2. Methods

### 2.1 PS-OCT imaging

3D volume scans were recorded using a custom-built spectral domain PS-OCT system [20]. It is based on a Michelson interferometer and uses polarization-maintaining (PM) fibers and a (single) circular polarized input state incident onto the eye. The sampling beam is generated by a superluminescent diode with a center wavelength of 860 nm, a bandwidth of 60 nm, and an A-scan rate of 70 kHz

To counteract involuntary eye movements, the system contains an updated retinal tracker [21]. The correction signal for the tracking is obtained by a line scanning laser ophthalmoscope that scans the retina simultaneously at 60 Hz with a light beam at a wavelength of 786 nm. The PS-OCT system achieves an axial, optical resolution of 4.2 µm in tissue (the pixel resolution is 2 µm axially), a lateral resolution of ∼20 µm, and a sensitivity of 98 dB [20].

The acquisition time of a single data set was 4.5 s. Each subject was imaged three times (on two different study days) at a region of size 28° (x) by 21° (y) centered at the macula, which corresponds to an area of ∼8 × 6 mm^2^ on the retina of a standard eye. The resulting volume scans consist of 250 B-scans each containing 1024 A-scans.

### 2.2 Subjects

156 healthy volunteers were enrolled into the study. 6 of them had to be excluded due to poor OCT image quality, so that 150 eyes of 150 healthy volunteers remained. Exclusion criteria were: low signal to noise ratio, failure of layer segmentation leading to erroneous compensation or en-face maps at the wrong position, strong residual motion artefacts. Their age ranged from 21.1 to 78.9 years (mean ± SD = 48.5 ± 16.3 years) and 84 were female and 66 male. Furthermore, we analyzed PS-OCT data in a subgroup of 87 age-matched (Student’s t-test: p = 0.059) healthy eyes (mean ± SD = 60.33 ± 8.74) and 64 eyes of 64 early-stage glaucoma patients (after excluding 6 patients due to poor OCT image quality), aged 39.9 to 78.4 years (mean ± SD = 63.2 ± 9.5 years). 37 of the patients were female, 27 male. Glaucoma specialists of the Medical University of Vienna diagnosed the patients based on characteristic features of the optic nerve head (ONH) and a repeatedly abnormal 24-2 visual field with a mean deviation of −6 dB or better. Patients showing evidence of other ocular diseases were excluded from this analysis.

This study was approved by the local ethics committee and adhered to the tenets of the Declaration of Helsinki. Prior to study inclusion, every patient gave written informed consent.

### 2.3 Data analysis

#### 2.3.1 Processing of spectral domain PS-OCT data

OCT data of the two channels of the PS-OCT system were recorded for each individual B-scan and processed using a standard processing protocol of spectral domain volume data. The amplitude and phase of the corresponding OCT signals were retrieved. The protocol includes the normalization of the spectra, the subtraction of an averaged spectrum, the rescaling of spectra from wavelength to wave number space, the numerical dispersion compensation, the Fourier transform, and the compensation of PM fiber length mismatch, as described previously [22]. From the OCT amplitude and phase data, PS-OCT data such as retardation, optic axis orientation, degree of polarization uniformity (DOPU) and Stokes vectors were retrieved.

To reduce noise in the PS-OCT data, data points with intensity values below a threshold were excluded from further processing. The threshold is determined by the noise measured in the central B-scan: the pixels of the central B-scans are sorted by intensity value, and the mean ( $\bar{I}$ ) and standard deviation ( $\sigma_{I}$ ) of the intensity of the 80% darkest pixels are calculated (assuming that the signal from the retina is contained in the 20% brightest pixels). In the first pixel row, the threshold equals $\bar{I}+7\cdot \sigma_{I}$ . This value decreases linearly in depth by a factor (corresponding to 0.08% of the standard deviation) per pixel to account for the decrease of noise with depth known from SD-OCT imaging. For detection of the retinal pigment epithelium (RPE), the DOPU was calculated using a kernel size of 10 × 10 pixels (corresponding to approximately 20 µm [axial] × 78 µm [lateral]), as described earlier [23]. A graph-based segmentation method (The Iowa Reference Algorithms, Retinal Image Analysis Lab, Iowa Institute for Biomedical Imaging, Iowa City, IA) [24,25] was used to segment inter-retinal layers similar to our previous work [26]. To determine the center of the fovea that is needed in the subsequent analysis, we used the outcome of the RNFL segmentation. In a first approximation, we defined the center of mass of the central area around the fovea where zero RNFL thickness is measured as foveal center.

#### 2.3.2 Compensation of anterior birefringence

As stated above, the PS-OCT system uses a light beam with a well-defined circular polarization state. However, after passing through birefringent structures (such as the cornea and the RNFL), the beam is no longer circularly polarized, which results in distorted polarization measurements of deeper structures such as HFL. Thus, the effects of anterior birefringent structures need to be compensated for to retrieve un-distorted polarization sensitive information of HFL. This can be done by using an adapted numerical algorithm [26] that initially was described for the compensation of the cornea alone [14]. The algorithm relies on a measurement of retardation and axis orientation at a reference surface that lies anterior to the structures of interest. Using this reference measurement a subsequent correction of deeper structures is performed without loss of information. To compensate for the cornea and the RNFL, a reference that lies posterior to RNFL and anterior to the HFL was chosen. Thereby, we took the brightest pixel within the depth range between the interface RNFL-GCL and the interface INL-OPL (red area in Fig. 1(a)).

#### 2.3.3 Generation of retardation and axis orientation en-face maps

After applying the compensation algorithm, retardation and axis orientation values were retrieved from a strong backscattering and polarization-preserving layer posterior to HFL, mainly the boundary between inner and outer photoreceptor segments (IS/OS, marked in blue in Fig. 1(a)). Note that the RPE is a strongly backscattering but depolarizing layer and pixels originating from this layer need to be excluded from this analysis because otherwise these distort the results. The segmentation algorithm mentioned above uses the OCT intensity data and already delivers a segmentation line for IS/OS. Since this line was not always exactly following the IS/OS, we corrected the segmentation in the following way. First, pixels originating from the depolarizing RPE (determined by low DOPU values) were removed. Then for each A-scan, the brightest pixel in a small neighborhood of the original segmentation line was determined and used as the corrected position of the segmentation line.

To further reduce noise, 5 pixels in depth (corresponding to ∼10 µm) around this layer (and with intensity values that lay above the initial intensity threshold) were averaged and retardation and axis orientation en-face maps were generated.

As shown previously [27], standard averaging of retardation values leads to an artificial offset because the retardation values are always positive. Thus, we used the same approach as outlined in [27] and performed averaging of the Stokes vector elements (Q,U,V) that are derived from the OCT amplitude and phase data. The averaged retardation $\delta_{\mathrm{avg}}$ was derived after normalization of the Stokes vector elements using the formula $\delta_{\mathrm{avg}}=\frac{1}{2}\cos^{-1}\left(\langle \frac{Q}{I}\rangle_{N}\right)$ , where *Q* and *I* are components of the Stokes vector (with *I* being the intensity). The angled brackets correspond to the mean, and $\left(\langle \frac{Q}{I}\rangle_{N},\langle \frac{U}{I}\rangle_{N},\langle \frac{V}{I}\rangle_{N}\right)=\frac{\left(\langle \frac{Q}{I}\rangle ,\langle \frac{U}{I}\rangle ,\langle \frac{V}{I}\rangle \right)}{\sqrt{\langle \frac{Q}{I}\rangle^{2}+\langle \frac{U}{I}\rangle^{2}+\langle \frac{V}{I}\rangle^{2}}}$ are the normalized components of the Stokes vector [27].

#### 2.3.4 Determination of HFL extension

**Fig. 2.**
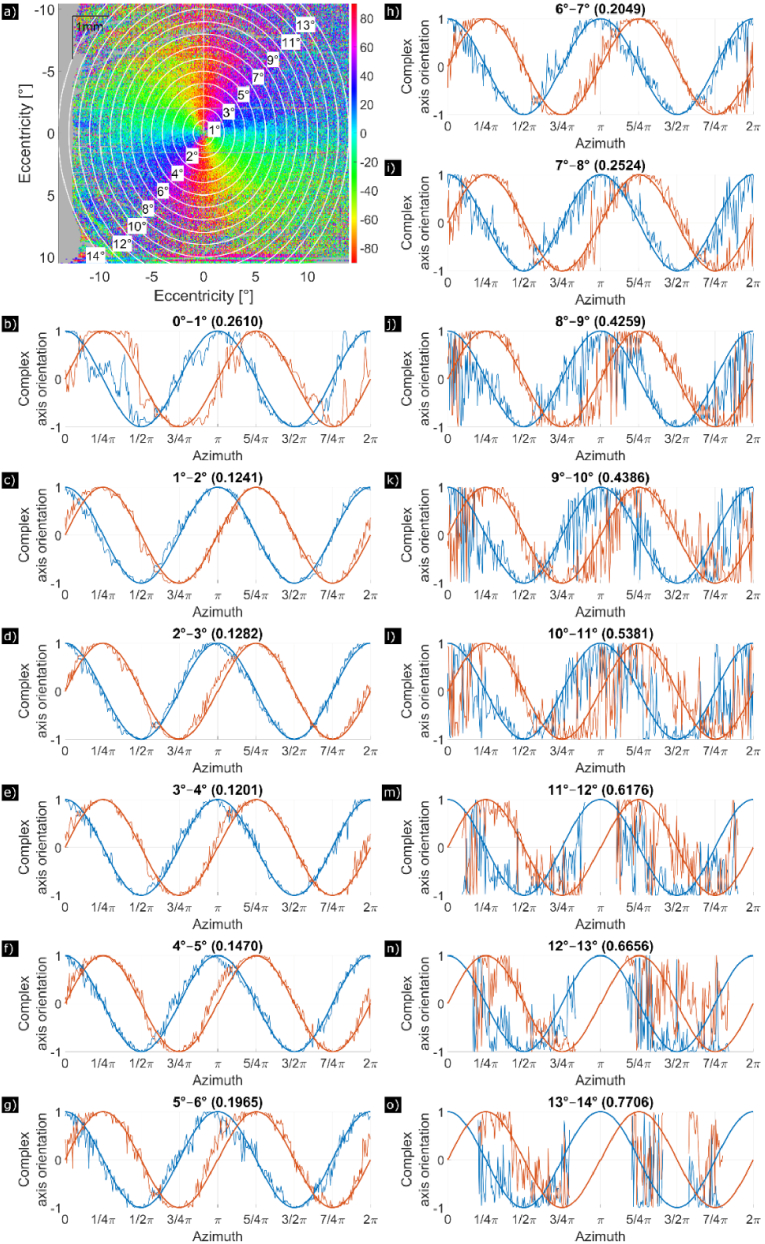
Axis orientation of HFL measured at IS/OS of a 30-year-old man with no evidence of eye diseases. (a) En-face map of axis orientation in a 28° (x) × 21° (y) field of view. (b) to (o) Axis orientation plotted in complex space, split up into real (blue) and imaginary (red) part, where 0° azimuthal angle ϕ is located at the white vertical line in a). The axis orientation is measured counterclockwise along concentric rings with a thickness of 1° of each annulus. Each panel from (b) to (o) shows the evolution of the axis orientation around the fovea for different annuli radii (with (b) being the center). The theoretically expected axis orientation in presence of HFL is plotted with a bold line, the measured axis orientation with a fine line. On top of each panel, the number in brackets denotes MD, which serves as a measure of the presence of the Henle fibers.

Previous work showed that the axis orientation measured by PS-OCT is a very sensitive indicator for the presence of birefringent tissue [26]. Here we want to use this observation for determining the lateral extension of HFL. Henle’s fibers are emerging from the fovea and are oriented radially. The fiber orientation is thereby indicated by the (slow) axis orientation measured by PS-OCT. In a complete circle that is centered on the fovea, the axis orientation increases linearly with the azimuth angle ϕ (cf. Fig. 1(e)). Because of the algorithm used for determining the axis orientation, the value range lies between −90° to +90° and the algorithm cannot differentiate between fiber orientations that are separated by 180°. Thus, for a full 360° circle of the azimuth angle, two full oscillations of the axis orientation can be observed (cf. Fig. 1(e) and Fig. 2(a)).

Here we propose to use this linear relationship to estimate the lateral extension of HFL. For that we investigate at which eccentricity from the fovea this linear relationship between axis orientation and azimuth angle no longer dominates. This was done as follows: first we generated 14 annuli with increasing radius (step size of 1°) around the fovea each with a radius width (larger radius minus smaller radius of the annulus) of 1° (cf. Fig. 2(a)). Then each annulus was divided into segments of 1° width for each integer value of the azimuth angle ϕ (in total 360 segments for each annulus). For averaging and for further processing we need to consider the cyclic nature of axis orientation. Thus, we transformed the axis orientation θ into a complex number *Z* that is defined in complex space on a unit circle using $Z(\theta )=1\cdot e^{i\cdot \theta }$ . In each segment, *Z* was averaged to reduce noise. Finally, plots of the real and imaginary parts of Z(θ) as a function of azimuth angle ϕ (or each segment) are generated. This yields the functions ReZ(ϕ) and ImZ(ϕ) , respectively and was done for all 14 annuli centered around the fovea (cf. Fig. 2(b)–(o)).

In the case of a perfect linear relationship between axis orientation and azimuth angle (corresponding to an ideal and regular Henle pattern), ReZ(ϕ) and ImZ(ϕ) will perform two full oscillations when the azimuth angle is varied from 0° to 360°. Therefore, we generated these ideal functions and determined for each azimuth angle the distance between the ideal function and the measured values. The mean (absolute) distance or mean deviation (MD) for all data points served as a measure of the presence of HFL. When no retardation is introduced (no presence of Henle fibers), a completely random axis orientation will be observed and a MD of 1.3 will be measured. As the transition from presence of Henle fibers to non-presence of Henle fibers can be gradual, we empirically defined a cut-off value of 0.65 for the MD, above which we assume no presence of HFL.

However, to optimize this analysis, the axis orientation values must be corrected in four ways as follows:

First, our method provides only a relative axis orientation [22] with an unknown effect that can vary over time due to minuscule deviations in the system and depending on the rotational positioning of the individual subjects during the measurement. Therefore, for each data set, before the following corrections, axis orientation was rotated (by adding a constant value to all measured axis orientation values) in such a way that at 0° azimuth angle the corresponding axis orientation value is −90°. To determine the required rotation angle, the “jump” from +90° to −90° was identified in an annulus around the fovea with a radius between 1° and 2° (cf. Fig. 2(c)).

Second, our initial method to determine the center of the fovea is only a first approximation. However, the choice of this position has an influence on the observed linear relationship between azimuth angle and measured axis orientation. Thus, the position of the center was iteratively corrected as follows: in the en-face map, each pixel in a window of 31 × 31 pixels around the original center was set as a new (potential) center. The MD was calculated in an annulus with a radius between 1° and 2° eccentricity (cf. Fig. 2(c)) around all these new centers. The set pixel yielding minimal MD was then used as a new center. The process was repeated until the center position as well as the MD did not change anymore.

Third, the compensation of the birefringence of anterior structures may be incomplete in some cases, resulting in an irregular retardation pattern (distorted “donut shape”), as can be observed in Fig. 1(c). Consequently, this leads to a non-linear increase of axis orientation with azimuth angle ϕ . To correct this, the en-face retardation and axis orientation maps were compensated repeatedly using the original values of retardation and axis orientation obtained at the reference tissue (GCL, IPL, INL) as the reference, but with two separate offsets added to the retardation and axis orientation. Both offsets were ranging from −5° to +5° with increments of 1°. Similar to the second correction, the offset combination yielding the most complete compensation is determined by finding the minimum MD in an annulus from 1° to 2° eccentricity.

Fourth, the fovea position is adapted again using the same method as explained above.

**Fig. 3.**
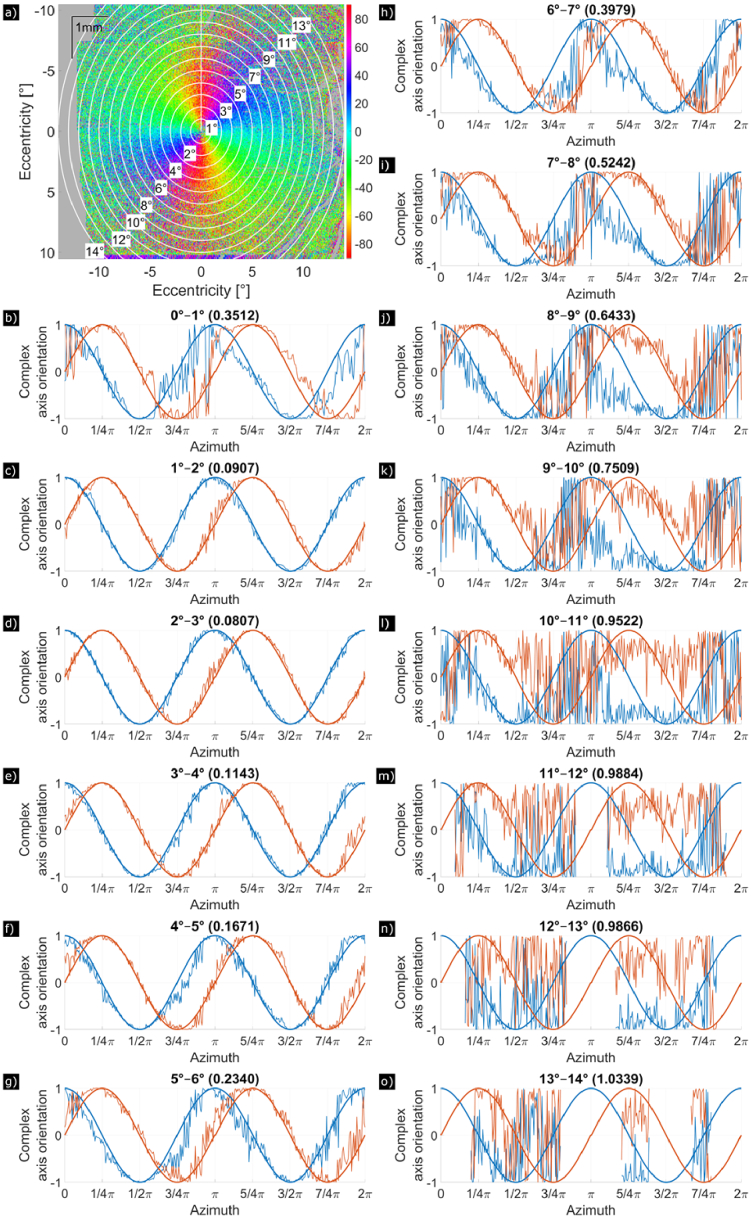
Axis orientation of HFL measured at IS/OS of a 65-year-old man with primary open angle glaucoma. (a) En-face map of axis orientation in a 28° (x) × 21° (y) field of view. (b) to (o) Axis orientation plotted in complex space, split up into real (blue) and imaginary (red) part, where 0° azimuthal angle ϕ is located at the white vertical line in a). The axis orientation is measured counterclockwise along concentric rings with a thickness of 1° of each annulus. Each panel from (b) to (o) shows the evolution of the axis orientation around the fovea for different annuli radii (with (b) being the center). The theoretically expected axis orientation in presence of HFL is plotted with a bold line, the measured axis orientation with a fine line. On top of each panel, the number in brackets denotes MD which serves as a measure of the presence of the Henle fibers.

After those corrections, the analysis in the various annuli could be performed as described. Figure 2 and Fig. 3 show an example of this analysis for a healthy volunteer and a glaucoma patient, respectively. Panel (a) shows the axis orientation en-face map with the annuli placement from 1° to 14° eccentricity. For each of the 14 annuli respectively, panels (b) to (o) plot the real and imaginary components of the ideal Henle pattern (bold) and of using the measured Z(θ) in dependence of the azimuthal angle ϕ , where ϕ=0 corresponds to the white vertical line and the azimuthal angle runs counterclockwise.

For each subject, three data sets were available. For further analysis, we only included one data set per subject that yielded a minimum MD between 1° and 8° eccentricity. This automatically excluded data sets with erroneous segmentations or corneal birefringence compensations.

#### 2.3.5 Circumferential average of retardation

**Fig. 4.**
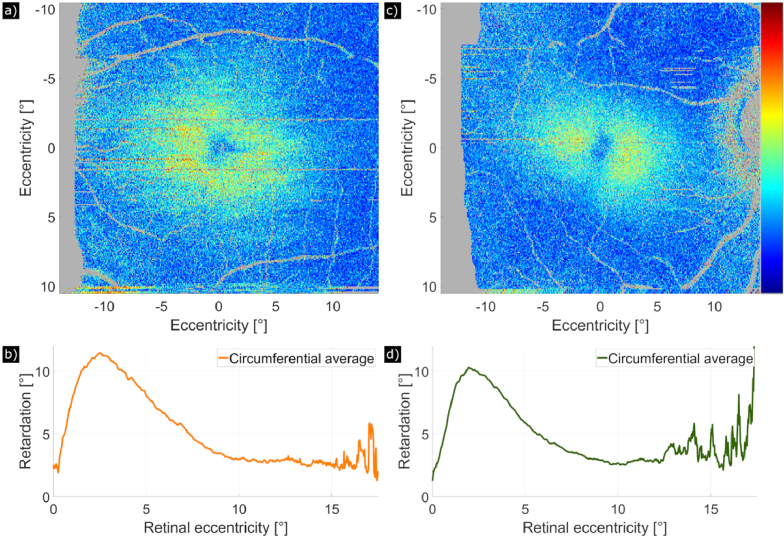
Retardation introduced by HFL and measured at IS/OS with full correction, (a) En-face map of retardation of a 30-year-old healthy man in a 28° (x) × 21° (y) field of view. (b) Corresponding circumferential average around the determined foveal center of the healthy volunteer plotted against retinal eccentricity. (c) En-face map of retardation of a 65-year-old man with primary open angle glaucoma in a 28° (x) × 21° (y) field of view. (d) Corresponding circumferential average of the glaucoma patient plotted against retinal eccentricity. *Colorbar: 0° to 25°*.

The compensated retardation data (including all corrections) for each subject was smoothed by averaging the Stokes vectors in windows of size 9 × 9 pixels (corresponding to approximately 18 [axial] × 70 [lateral] µm^2^) and the smoothed retardation was averaged circumferentially around the determined foveal center. Examples of a healthy volunteer and a glaucoma patient are shown in Fig. 4. Figure 4(a) shows the same data set as displayed in Fig. 1(c) but after applying the various correction steps. Thus, a more radially symmetric retardation pattern can be observed. The quantitative evaluation by averaging the retardation values circumferentially around the determined foveal center yields maximum retardation values of 11.5° and 10.3° for the healthy volunteer and the glaucoma patient, respectively (cf. Fig. 4(b) and (d)).

## 3. Results

In Fig. 2, the axis orientation Henle pattern is shown for a 30-year-old male healthy volunteer. Using our cut-off at an MD of 0.65, Henle fibers extend up to 12° (Fig. 2(m)). The Henle pattern of the 65-year-old male glaucoma patient in Fig. 3 extends to only 9° (Fig. 3(j)). In the following analysis, age and glaucoma are investigated as possible factors that influence HFL extension.

**Fig. 5.**
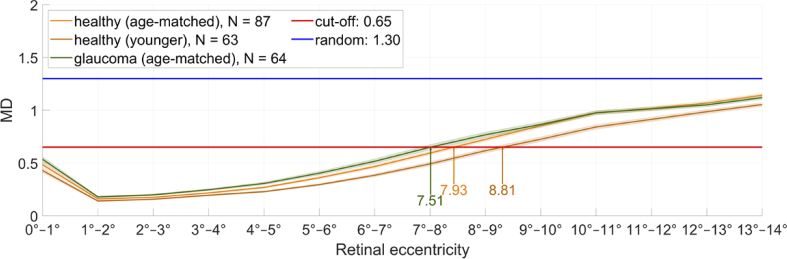
MD plotted against the retinal eccentricity with the standard error (shaded) for healthy volunteers (orange: age-matched, dark orange: subjects younger than 39 years) and glaucoma patients (green) with the cut-off distance at an MD value of 0.65. Healthy volunteers have been separated by age to match the on average older glaucoma patients. Glaucoma patients have a shorter HFL extension (7.51°) than age-matched healthy volunteers (7.93°) on average. The younger healthy volunteers on average have a larger HFL extension (8.81°) than the older healthy volunteers.

Figure 5 shows the MD in each annulus, averaged over healthy volunteers (orange) and glaucoma patients (green). The healthy volunteers are split up by age to match the age of the glaucoma patients (dark orange: healthy volunteers younger than 39 years). The shaded area corresponds to the standard error between the subjects. In the very center of the fovea, the axis orientation is not defined. Consequently, MD shows increased values at the central disk of the fovea. Starting from the minimum MD value at 1°–2° annulus, the MD increases towards the periphery for all subjects.

The glaucoma patients tend to have a shorter extension of HFL than the healthy volunteers, and the older healthy volunteers tend to have a shorter extension of HFL than the younger healthy volunteers: in case of the glaucoma patients, the MD curve intersects the cut-off of 0.65 at 7.51° retinal eccentricity from the fovea on average, whereas for the age-matched healthy volunteers, on average, the intersection is at a retinal eccentricity of 7.93°. For the younger healthy volunteers, the intersection is at 8.81° retinal eccentricity, on average.

**Fig. 6.**
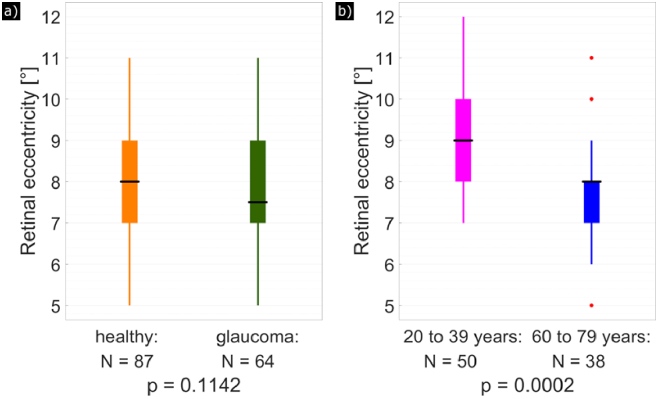
Box plot diagrams comparing the retinal eccentricities of HFL extension. (a) Age-matched healthy volunteers (orange) compared to glaucoma patients (green). (b) Young healthy volunteers (magenta) compared to old healthy volunteers (blue). The edges of the boxes correspond to the 25^th^ (P_25_) and 75^th^ (P_75_) percentiles, the median is drawn as a black horizontal line, the whiskers extend to the most extreme data points that are no outliers, and outliers (points larger than P_75 _+ 1.5·(P_75_−P_25_) or smaller than P_25_−1.5·(P_75_−P_25_)) are drawn as red dots. Number of subjects per study group as well as the p-value (Student’s t-test) are specified below each box plot.

The difference between the retinal eccentricity of age-matched healthy volunteers and glaucoma patients (determined by a cut-off MD of 0.65) is also shown in the box plot diagram in Fig. 6(a). However, at 5% significance level (Student’s t-test), the difference is not statistically significant (p = 0.1142). In Fig. 6(b), the youngest healthy volunteers (between 20 and 39 years old) and the oldest healthy volunteers (between 60 and 79 years old) were compared in a box plot diagram. With a p-value of 0.0002, the two groups are significantly different.

**Fig. 7.**
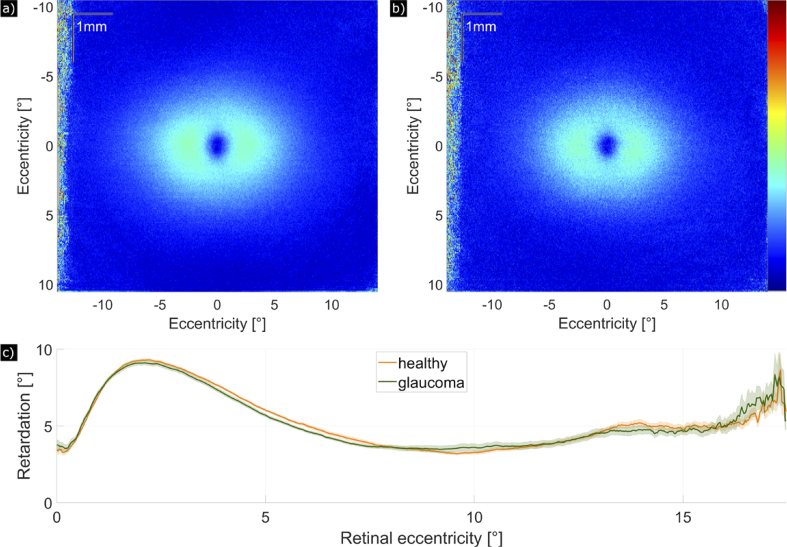
Retardation induced by HFL averaged over age-matched healthy volunteers and glaucoma patients. (a) Retardation en-face map averaged over 87 healthy volunteers, showing the annular shape of elevated retardation due to the Henle fibers. (b) Retardation en-face map averaged over 64 glaucoma patients with a very similar pattern to healthy eyes. (c) Circumferentially averaged retardation plotted against retinal eccentricity with standard error (shaded). Healthy volunteers (orange) and glaucoma patients (green) show a nearly identical curve. *Colorbar: 0° to 25°*.

The retardation introduced by HFL and averaged over age-matched healthy volunteers and glaucoma patients is shown in Fig. 7(a) and (b) respectively. In Fig. 7(c), the circumferential average of retardation is plotted against eccentricity from the fovea, averaged over age-matched healthy volunteers (orange) and glaucoma patients (green) and including the standard error across subjects. Retardation at the fovea is small (< 4° on average for both groups) and increases quickly to 9° at an eccentricity of 2° from the fovea, corresponding to the annular shape (“donut”). Healthy volunteers and glaucoma patients appear to have a very similar retardation pattern, but the retardation appears to be slightly higher for healthy volunteers in the region of about 2° to 7.5° retinal eccentricity.

**Fig. 8.**
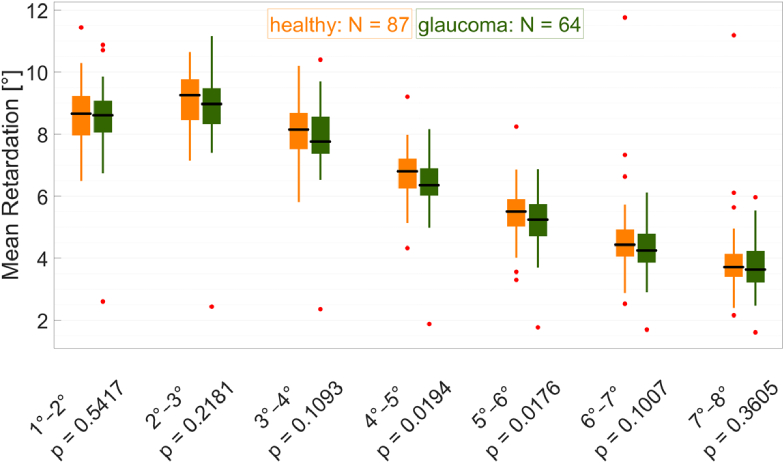
Box plot diagrams comparing age-matched healthy volunteers (orange) to glaucoma patients (green) for each annulus (retinal eccentricity from the fovea). The edges of the boxes correspond to the 25^th^ (P_25_) and 75^th^ (P_75_) percentiles, the median is drawn as a black horizontal line, the whiskers extend to the most extreme data points that are no outliers, and outliers (points larger than P_75 _+ 1.5·(P_75_−P_25_) or smaller than P_25_−1.5·(P_75_−P_25_)) are drawn as red dots. The p-values (Student’s t-test) are specified below each box plot.

To compare the retardation between healthy volunteers and glaucoma subjects as well as between younger healthy subjects (20 to 39 years) and older healthy subjects (60 to 79 years), we introduced four different measures: the maximum retardation (usually occurring at around 2° eccentricity from the fovea), the eccentricity of the maximum retardation, the retardation area under the curve (AUC) between 1° and 8° eccentricity, and the mean retardation per annulus (1°–2° to 7°–8°). For each of these measures, we performed Student’s t-test, and box plot diagrams of the mean retardation per annulus along with the corresponding p-values for the comparison between healthy age-matched volunteers (orange) and glaucoma patients (green) are shown in Fig. 8.

The difference between younger and older subjects was not significant for any of these measures: maximum retardation (p = 0.8723), maximum retardation eccentricity (p = 0.9563), retardation AUC (p = 0.4208), mean retardation (p = 0.7045, p = 0.7045, p = 0.7045, p = 0.7045, p = 0.7045, p = 0.7045, and p = 0.7045 for annuli 1°–2°, 2°–3°, 3°–4°, 4°–5°, 5°–6°, 6°–7°, 7°–8°, respectively).

Comparing healthy volunteers to glaucoma patients, the differences were not significant for the following measures: maximum retardation (p = 0.7618), maximum retardation eccentricity (p = 0.6307), retardation AUC (p = 0.6104), mean retardation (p = 0.5417, p = 0.2181, p = 0.1093, p = 0.1007, and p = 0.3605 for annuli 1°–2°, 2°–3°, 3°–4°, 6°–7°, 7°–8°, respectively). However, glaucoma seems to have a statistically significant effect on the mean retardation in the annuli 4°–5° and 5°–6° (p = 0.0194 and p = 0.0176, respectively).

## 4. Discussion

Due to its fibrous nature, Henle fibers exhibit form birefringence that can be assessed by PS-OCT. Thereby, the axis orientation serves as a very sensitive indicator to detect the presence of birefringent structures. Based on this we introduced a new quantitative method to determine the lateral extension of HFL. The method relies on the very regular arrangement of Henle fibers and the corresponding radial extension from the foveal center. Considering the anatomical structure of Henle fibers [1], this is an assumption that is certainly fulfilled in the healthy eye.

**Fig. 9.**
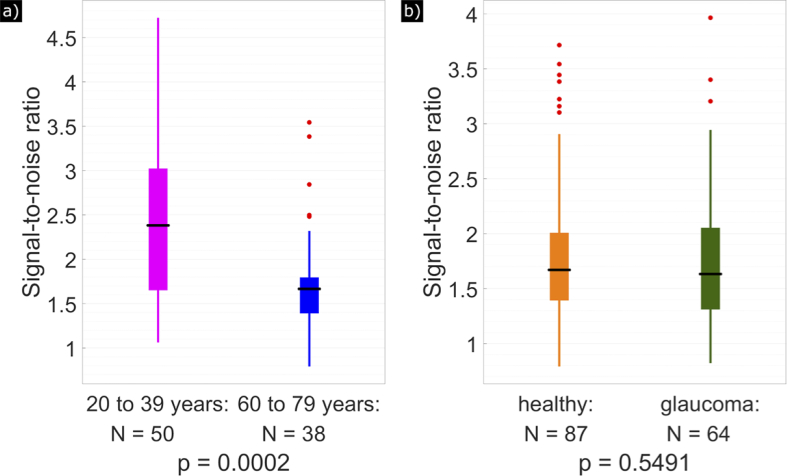
Box plot diagrams estimating a potential bias introduced by signal strength. (a) Comparison of 50 younger healthy volunteers (magenta) to 38 older healthy volunteers (blue). (b) Comparison of 87 age matched healthy volunteers (orange) to 64 glaucoma patients (green). The edges of the boxes correspond to the 25^th^ (P_25_) and 75^th^ (P_75_) percentiles, the median is drawn as a black horizontal line, the whiskers extend to the most extreme data points that are no outliers, and outliers (points larger than P_75 _+ 1.5·(P_75_−P_25_) or smaller than P_25_−1.5·(P_75_−P_25_)) are drawn as red dots. The p-values (Student’s t-test) are specified below each box plot.

The introduced parameter, mean deviation MD, considers the cyclic character and associated phase wrappings of the axis orientation measurements. The cut-off value of 0.65 was empirically set to approximately half of the value that is obtained for MD when a completely random axis orientation is measured. Although this cut-off value is somewhat arbitrary, and Henle fibers likely extend beyond this limit (although increasingly difficult to discern from noise at higher MD values), a closer inspection of Fig. 5 indicates that this selection seems reasonable because it provides an optimum differentiation between the study populations. Thereby, we found a relationship between fiber extension and age of the subject, with elderly subjects showing reduced extension of HFL compared to younger subjects. This observation is possibly associated with normal aging and normal loss of neuronal tissue or photoreceptors [28]. In addition, there might be a bias because older subjects on average obtained a lower image quality than younger subjects. To test this, we determined the signal-to-noise ratio (SNR) for each data set the following way: as described in the methods section, PS-OCT data points were excluded if their intensity value was below a depth-dependent noise threshold. For each pixel in the data set used to calculate the axis orientation and retardation en-face maps at IS/OS, the ratio between the intensity value and the noise threshold was calculated and averaged. According to Student’s t-test between healthy volunteers younger than 40 years and healthy volunteers aged 60 years or older, the difference in SNR was significant at 5% (p = 0.0002, Fig. 9(a)). Thus, the observed significant difference in HFL extension between younger and older subjects may simply be caused by the different SNR in our measurements. To confirm this, we performed a regression analysis for all healthy subjects between the SNR observed at the corresponding en-face plane and the measured HFL extension. This analysis showed a significant correlation (p = 0.0021) indicating that the SNR needs to be considered when comparing the HFL extension between groups. However, there might be a possibility to adapt the MD cut-off value to account for varying SNR.

Figure 9(b) shows the box plot diagrams comparing the SNR of age-matched healthy volunteers to glaucoma patients. The p-value of 0.5491 indicates that the image quality of age-matched healthy and glaucoma data sets is equivalent. Thus, the significant difference in mean retardation between 4° and 6° between both groups is independent of SNR. Although we only included early stages of the disease, this might indicate that the neuronal tissue of HF is already affected. Further studies including later-stage glaucoma cases are needed to confirm this effect.

To test the repeatability of the method, we determined the retinal HFL extension using three different measurements each in a subset of 65 healthy volunteers and 19 glaucoma patients and calculated the standard deviation for the determined HFL extension. Subjects were only included into this subset analysis when three measurements with good image quality, good layer segmentation, good compensation results, and no major motion artifacts were available. The standard deviations ranged from 0° to 2.0817° (0.7891° on average).

As the light needs to penetrate anterior structures of the eye like the cornea and, at larger eccentricity from the fovea, the RNFL to reach HFL, we need to compensate for birefringence introduced by these [14]. Typically, this is done by retrieving retardation and axis orientation at an interface posterior to RNFL but anterior to HFL. We noticed however, that this procedure did not always result in the typical “donut shape” pattern in the en-face maps of the retardation (and the linear increase of axis orientation with azimuth angle) introduced by HFL. Using numerical simulations, we found that already very small deviations in the order of a few degrees (experimentally we frequently found deviations of ±1°) from retardation and axis orientation values that optimize the “donut shape” results in a severe distortion of the “donut pattern”. We hypothesize that imprecise measurements of the anterior retardation and axis orientation values are the main cause for our observation. These are most likely due to the noisy measurement that is associated with the relatively low signal to noise ratio that is typically observed at the layers posterior to RNFL and anterior to the IS/OS. To automatically correct for this, we used a method based on minimizing the MD value in an annulus with an inner radius of 1° and outer radius of 2°. This choice is motivated by the high retardation introduced by HFL measured at this eccentricity and in principle any annulus up to 5° eccentricity (cf. Fig. 7) might be used as well, most likely leading to similar results.

Another critical issue for our method is the choice of the foveal center because this influences the linearity between axis orientation and azimuth angle. By calculating the MD for the 1°–2° annulus and looking for a minimum, while varying the location of the center within the central macula, we have found a new and automatic method for determining the center of the Henle fiber pattern. Our approach extends previous concepts that were as well based on the polarization properties of Henle fibers but required manual selection of the center [29] or were used for determining the locus of fixation [30]. The question remains if the location of the center of Henle fiber pattern coincides with the central fovea defined by the location of highest cone density. Previous work using adaptive optics showed for example that the location of fixation does rarely coincide with the location of highest cone density [31]. However, to answer this questions, additional measurements with adaptive optics assisted instruments are required.

Figure 7 shows the typical “donut pattern” of the retardation that is found by averaging over all healthy and glaucoma subjects, respectively. By averaging the data circumferentially, we determined that the highest retardation values can be found around 2° eccentricity from the fovea. This is comparable to previous work [16] that included 3 subjects and where the highest value was observed between 1.5° and 2.25°. In our study we found at the maximum of the retardation distribution a value of 9° (21.5 nm), which is slightly lower than the corresponding values of previous work (10.2° to 11.5° single-pass retardation). However, this slight deviation can be explained by the individual variability.

Noticeable is a slight asymmetry of the retardation pattern (cf. Fig. 7 (a) and (b)), where the area of high retardation has a larger lateral extension in the nasal and temporal direction as compared to the inferior and superior one. This observation is most likely linked to an asymmetry that can be observed in cone density [32]. Similar to the asymmetry in HFL, a higher cone density can be observed in the nasal-temporal direction than in the inferior-superior direction.

In our study population we found a large variability in radial retinal extensions of HFL between 5° and 12° from the foveal center. As the SNR has an influence on the HFL extension that we measure using MD, with lower values in the case of low SNR, we are likely underestimating the true extension of HF in these cases. In a standard eye, our values correspond to a distance from the fovea of 1.4 mm to 3.4 mm, or a circular area of 6.4 mm^2^ to 36.9 mm^2^. Previous work used standard OCT imaging in combination with off-center pupil illumination to measure HFL thickness [4]. The extension of HFL was then defined as area where the measured HFL thickness exceeds the mean thickness of HFL (∼26.5 µm) in the observed area of 5.6 × 5.6 mm^2^. Thereby, a HFL extension of 10.771 ± 0.574 mm^2^ was reported, which lies in the range of our values but is certainly at the lower end. This discrepancy arises from the different definitions that are used to determine the presence of HFL. In our case we used the MD value that originates from the regular arrangement of HFL while the previous report used a HFL thickness threshold. As the measured HFL extension based on the MD is larger (specifically in the case of high SNR), a regular arrangement of HFL can still be detected in areas where the HFL thickness lies below the average HFL thickness.

Compared to histology where eyes were sectioned to determine the length of individual HF and ganglion cell displacement, our results are in good agreement. In nasal direction at about 3.4 mm from the foveal center, the ganglion cell displacement was found to be zero [3], which corresponds to the widest HFL extension found in our study. Temporal to the center of the fovea, ganglion cell displacement was detected up to 4.5 mm, which is slightly larger than our values. Our results are comparable specifically if we consider that lower SNR leads to a lower measured HFL extension.

Previous work investigating polarization effects induced by HFL using SLP found a reduction of retardation with age [19]. Interestingly, we did not find a related decrease of retardation: none of the retardation-related parameters we analyzed showed a significant reduction with age. The differences between our observations and those of the SLP-based study might be caused by the different measurement and data evaluation methods, and further studies involving a direct comparison of the results of both methods obtained in the same set of subjects might be required to solve this issue.

Finally, we want to point out that disruptions of the regular HFL pattern occur in the presence of diseases affecting the macula such as AMD [18]. This can be used to obtain additional information on structural tissue changes. Although our method relies on a regular arrangement of Henle fibers, the algorithm will find corresponding minima of the MD even in the case of distorted patterns and will most likely succeed at least in not very severe cases. Nevertheless, future work is needed to test the applicability of the method in these diseases.

## 5. Conclusion

We defined a new measure to determine the presence of Henle fibers at various eccentricities from the fovea. This allowed for a comparison of the extension of HFL between healthy age- matched subjects and early-stage glaucoma patients. The measure is based on optic axis orientation (retrieved using polarization-sensitive OCT), which shows a radial pattern due to the HFL’s structure. As this metric depends on the SNR it is important to include a SNR analysis for a comparison between study groups. We quantitatively described the “donut-shaped” pattern of elevated retardation in the fovea and found this metric rather independent from the SNR. Maximum retardation was found at about 2° eccentricity and reached values of 9° on average. Various metrics of the retardation pattern were found to be independent from age. Finally, the mean retardation between 4° and 6° eccentricity in the glaucoma group differed significantly from an age-matched healthy group, which implies that the HFL of these patients might already be affected by the disease at this stage. We expect this method to be applicable to other retinal diseases that affect the macula to monitor structural changes of HFL and associated retinal tissue.

## Data Availability

Data underlying the results presented in this paper are not publicly available at this time but may be obtained from the authors upon reasonable request.
